# A psychometric evaluation of the Female Sexual Function Index in women treated for breast cancer

**DOI:** 10.1002/cam4.4516

**Published:** 2022-02-08

**Authors:** Genevieve A. Kieseker, Debra J. Anderson, Janine Porter‐Steele, Alexandra L. McCarthy

**Affiliations:** ^1^ School of Health and Rehabilitation Sciences The University of Queensland St Lucia Queensland Australia; ^2^ Faculty of Health The University of Technology Sydney Ultimo Australia; ^3^ Choices Cancer Support Program Wesley Hospital Brisbane Queensland Australia; ^4^ School of Nursing, Midwifery and Social Work The University of Queensland, and Mater Research Institute St Lucia Queensland Australia

**Keywords:** breast cancer, factor analysis, Female Sexual Function Index, psychometrics, sexual function

## Abstract

**Background:**

We aimed to determine the psychometric properties and factor structure of the 19‐item Female Sexual Function Index (FSFI) in 132 sexually active women previously treated for breast cancer.

**Methods:**

Confirmatory factor analysis explored three models: (a) second‐order six‐factor, (b) six‐factor, and (c) five‐factor models combining the desire and arousal subscales.

**Results:**

Results revealed excellent reliability for the total score (Cronbach's α = 0.94), and domain scores (all Cronbach's αs > 0.90), and good convergent and discriminant validity. The six‐factor model provided the best fit of the models assessed, but a marginal overall fit (Tucker–Lewis index = 0.91, comparative fit index = 0.93, root mean square error of approximation = 0.09). Exploratory factor analyses (EFA) supported a four‐factor structure, revealing an arousal/orgasm factor alongside the original pain, lubrication, and satisfaction domains.

**Conclusion:**

The arousal/orgasm factor suggests a “sexual response” construct, potentially arising from an underlying latent factor involving physical and mental stimulation in conceptualizations of arousal and orgasm in women treated for breast cancer. Finally, the EFA failed to capture an underlying desire factor, potentially due to measurement error associated with the small number of items (two) in this domain. Despite evidence that the FSFI has sound psychometric properties, our results suggest that the current conceptualizations of the FSFI might not accurately represent sexual functioning in women previously treated for breast cancer. Further research is required to elucidate the factors that influence desire, arousal, and orgasm in sexually active women in this population, and the reasons underlying sexual inactivity. Practical and theoretical implications for FSFI use in this population are discussed.

## Graphical abstract

The Female Sexual Function Index (FSFI) has sound reliability and validity in sexually active women treated for breast cancer. However, structural validity evaluations via confirmatory and exploratory factor analyses indicate that the current conceptualizations of the FSFI may not accurately represent sexual functioning in this population.

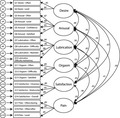

## 1 INTRODUCTION

Up to 77% of women with breast cancer report sexual dysfunction during and after treatment.^1^ Cancer treatment can damage the structure and function of endocrine, dermal, neural, and blood vessel tissues, which results in pain or inhibits desire.^2^ Diagnosis and treatment can also trigger psychological concerns such as anxiety, depression, fear of recurrence, and body image alterations that can negatively affect sexual activities and responses.^3^, ^4^ To support these women through the physical, psychological, and emotional changes in sexual function, accurate measures are necessary to identify those who need further support.

The 19‐item Female Sexual Function Index (FSFI) is the current gold standard measure of female sexual function in research and practice.^5^ The FSFI assesses sexual function in six domains: desire, arousal, lubrication, orgasm, satisfaction, and pain, with a composite total score representing an overall sexual function.^5^ While previous psychometric evaluations of the FSFI typically report good reliability (Cronbach α ≥ 0.82) and high discriminant and convergent validity,^5^, ^6^ a review by Neijenhuijs et al. highlighted inconsistent evidence regarding the structural validity of the 19‐item FSFI.^7^ Specifically, of the 28 reviewed studies that reported indices of structural validity, nine supported a six‐factor model (i.e., the six domains), 12 supported a five‐factor model (i.e., merged desire and arousal domains), and seven supported a less‐than‐five‐factor model (i.e., multiple merged domains). With more evidence *against* rather than favoring the original six‐factor FSFI structure,^5^ there is a possibility that the structure of the FSFI is population‐specific. To this end, investigations of the FSFI’s factor structure in cancer populations revealed support for a five‐factor model,^8^ but a six‐factor model in breast cancer populations.^6^ Thus, our study seeks to contribute to the growing psychometric support of the FSFI by evaluating the reliability and validity, including the structural validity, of the 19‐item FSFI in a population of women previously treated for breast cancer.

Our group conducted a randomized controlled trial where women previously treated for breast cancer were assigned to receive usual care (control) or a structured lifestyle intervention (Women's Wellness After Cancer Program [WWACP]).^9^ This intervention was designed to address quality‐of‐life health needs after breast cancer treatment, in which sexual function concerns were also addressed. As previous evidence supports a high degree of FSFI acceptability in a sample of sexually active women with breast cancer,^6^ this index was chosen to assess changes in sexual functioning as a result of the WWACP. To account for the reported inconsistencies in the structural validity of the FSFI,^6^, ^7^, ^8^ several authors advise that clinical researchers who use this index should perform confirmatory factor analyses and report the factor structure obtained in their samples.^7^, ^10^ As such, prior to assessing intervention‐related changes in FSFI scores, it is first necessary to establish the structural validity of the FSFI in our sample. This investigation can help elucidate if the FSFI is population‐specific by accumulating evidence for or against the original six‐factor model in the population of interest. Additionally, determining the factor structure has important implications regarding the use of the FSFI in clinical and research settings, especially in the decision to merge, or not to merge, the subscales of desire and arousal.^10^ For example, the ability to distinguish deficits in desire, subjective arousal, and physical arousal might be more desirable for healthcare providers as they can use this information to provide treatment or support that is tailored to the individual.

Accordingly, to inform future studies with breast cancer samples, we sought to replicate and expand upon previous studies that supported either a five‐ or six‐factor model in cancer and breast cancer samples.^6^, ^8^ To do so, we tested three common and competing FSFI models via confirmatory factor analysis (CFA): the (a) the *second*‐*order six‐factor model* based on domain scores aggregating to form a total scale score,^5^, ^11^ (b) the *six*‐*factor model* based on empirical evidence supporting six latent variables,^5^, ^6^, ^11^, ^12^ and (c) the *five*‐*factor model* with merged arousal and desire domains.^5^, ^8^

## 2 METHOD

### 2.1 Participants

The data were obtained from a subset of participants enrolled in the WWACP.^9^ Our initial sample comprised 269 women (range = 34–74 years) treated for Stage I and II breast cancer in the last 5 years. No participant had metastatic or advanced cancer, inoperable or active loco‐regional disease. Twelve participants were excluded from further analyses due to non‐response and inadequate sampling (i.e., missing more than 50% of responses).

Furthermore, as the FSFI was previously validated in sexually active women, the 125 participants who reported “no sexual activity” (i.e., a score of zero) on any item were considered sexually inactive and excluded from further analyses. All subsequent analyses reported here, therefore, report results for the sample of 132 sexually active women. We employed this conservative approach as participants were not screened for the presence of sexual activity within the preceding four weeks. Additionally, this approach reduces the bias toward greater dysfunction by ensuring that low scores are non‐zero, given that the FSFI scoring algorithm assumes that zero scores represent low levels of sexual functioning.^5^, ^11^ Finally, only baseline FSFI data were included in this psychometric analysis to ensure naïve responses and reduce noise as a result of intervention‐related artifacts.

Ethical approval was granted by the Queensland University of Technology Human Research Ethics Committee (Approval No: 1300000335) in July 2013 and funded by a National Health and Medical Research Council (NHMRC) Partnership Grant (APP1056856). Local ethics approval was also gained from all participating hospitals and health services. Each participant provided written, informed consent prior to participation.

#### 2.1.1 Missing data analysis and solution

Of the 132 sexually active participants, 98 provided complete data, and all participants were adequately sampled (i.e., no participant was missing more than 50% of responses). All variables were adequately assessed, with 50 missing values comprising <2% of total responses. A non‐significant Little's missing completely at random test revealed no systematic patterns within the missing data, χ^2^ (*N* = 344) = 373.36, *p* = 0.133, indicating the acceptability of our planned inferences. The missing data values were estimated using the Expectation‐Maximization (EM) algorithm. This approach was appropriate as conventional Maximum Likelihood estimation approaches do not work well in the presence of missing data. Through 250 iterative cycles, the EM algorithm estimates the missing data based on the known information of the variables and variable relationships and then optimizes the parameters of the model to provide the best fit for the data.^13^, ^14^

### 2.2 Measures

The 19‐item self‐reported FSFI includes 15 items containing a zero‐scored option indicating no sexual activity. Otherwise, responses range on a 5‐point scale from 1 (*Never*) to 5 (*Always*) measuring six domains of sexual functioning: desire, arousal, lubrication, orgasm, satisfaction, and pain. This is consistent with conceptualizations of female sexual dysfunction in the Diagnostic and Statistical Manual of Mental Disorders 5th Edition (DSM‐5).^15^ Scores in each domain are summed and multiplied by a domain factor to produce a domain score (range = 0/1–6). Summed domain scores produce the total score (range = 2–36). Higher scores indicate higher sexual function.^5^ Table 1 displays the descriptive statistics including EM‐estimated missing values. Cut‐off scores indicate sexual dysfunction.^16^

**TABLE 1:** Descriptive statistics of FSFI domain and total scores of sexually active participants

	*N*	Mean (SD)	Cut‐off scores	Min.	Max.
Desire	132	2.65 (1.06)	<4.28	1.20	5.40
Arousal	132	3.38 (1.30)	<5.08	1.20	6.00
Lubrication	132	3.83 (1.57)	<5.45	1.20	6.08
Orgasm	132	3.78 (1.64)	<5.05	1.20	6.07
Satisfaction	132	3.83 (1.48)	<5.51	0.40	6.10
Pain	132	4.34 (1.64)	<5.04	1.20	6.00
FSFI total score	132	21.81 (6.73)	<26.55	7.20	34.50

Note

Minimum values <1.20 and maximum values >6 are due to Expectation‐Maximization estimation of missing values.

Abbreviations: FSFI, Female Sexual Function Index; SD, standard deviation.

### 2.3 Statistical analyses

The Statistical Package for the Social Sciences (SPSS) Version 27.0 was used to generate descriptive statistics, conduct assumption tests prior to conducting CFA, and generate estimates of internal consistency. The SPSS Analysis of Moment Structures (AMOS) module Version 26.0 was used in the CFA.

CFA examined the FSFI subscale structure using maximum likelihood model estimation, an appropriate method when Likert‐type items have more than three response categories and are not significantly (>1) differentially skewed.^17^ The FSFI has five response categories in sexually active women, and preliminary analyses revealed that all items had acceptable skewness (range from −1.00 to 0.76). Standardized regression estimates are reported for ease of interpretation. For the six‐factor and five‐factor models, factor scaling was utilized where one factor loading from each factor was set as 1. Factor scaling for the second‐order six‐factor model was achieved by fixing one item per factor to 1 in the first order and one domain to 1 in the second order. A sample size of 132 is appropriate as the significance rule and the participants‐to‐variables ratio were met.^18^

### 2.4 Main outcome measures

Model fit was assessed using the minimum discrepancy per degree of freedom criteria (χ^2^/df), Tucker–Lewis index (TLI), comparative fit index (CFI), root mean square error of approximation (RMSEA), and standardized root mean square residual (SRMR).^19^ The goodness‐of‐fit indices are described in Table 2. The Akaike information criterion (AIC) was used to compare fit among models, with smaller values indicating better fit.^20^ Squared multiple correlations (i.e., item communalities) and standardized regression weights (i.e., factor loadings) describe the model in detail.

**TABLE 2:** Goodness‐of‐fit indices

	Good fit	Marginal fit	Comments
χ^2^	*p* < 0.001	*p* < 0.001	Sensitive to large sample and model sizes
χ^2^/df criteria	<3.00^18^	<5.00^21^	<2.00 indicates a good fit^22^
TLI	>0.95^23^	>0.90^24^	Non‐normed fit index (<0 or >1) Unaffected by sample size^25^
CFI	>0.95^23^	>0.90^24^	Normed fit index (0–1)
RMSEA	<0.05^26^	<0.08^27^	Non‐centrality‐based fit index
SRMR	<0.08^23^		Average residual correlations

Abbreviations: CFI, comparative fit index; RMSEA, root mean square error of approximation; SRMR, standardized root mean square residual; TLI, Tucker–Lewis index.

## 3 RESULTS

### 3.1 Sample characteristics

Demographic and medical characteristics of the whole sample are provided in Table 3.

**TABLE 3:** Sample background demographic and medical characteristics

Variables	Sexually active (*n* = 132)	Excluded and sexually inactive (*n* = 137)	Total (*n* = 269)
	*n* (%) or *M* (SD)	*n* (%) or *M* (SD)	*n* (%) or *M* (SD)
Mean age (SD)	52.31 (8.14)	54.85 (8.26)	53.54 (8.28)
WWACP intervention (%)	57 (43.2)	71 (56.8)	128 (49.8)
Marital status (%)			
Married/de facto	122 (92.4)	77 (61.6)	199 (77.4)
Separated/divorced	6 (4.5)	22 (17.6)	28 (10.9)
Widowed	1 (0.8)	5 (4.0)	6 (2.3)
Single	2 (1.5)	20 (16.0)	22 (8.6)
Country of birth (%)			
Australia	85 (64.4)	85 (68.0)	170 (66.1)
Elsewhere	47 (35.6)	40 (32.0)	87 (33.9)
Educational attainment (%)			
Primary or junior school	10 (7.6)	10 (8.0)	20 (7.8)
Senior school	13 (9.8)	12 (9.6)	25 (9.7)
Trade qualification	29 (22.0)	30 (24.0)	59 (23.0)
University degree	52 (39.4)	38 (30.4)	90 (35.0)
Postgraduate degree	28 (21.2)	34 (27.2)	62 (24.1)
Cancer treatment (% ‘yes’)			
Surgery	132 (100)	124 (99.2)	256 (99.6)
Chemotherapy	94 (71.2)	86 (68.8)	180 (70.0)
Tamoxifen	66 (50.0)	37 (27.0)	103 (40.0)
Aromatase inhibitors	38 (28.8)	56 (40.9)	92 (35.8)

Abbreviations: SD, standard deviation; WWACP, Women's Wellness After Cancer Program.

### 3.2 Reliability

This scale has excellent reliability for the total score (Cronbach's α = 0.94), and domain scores (desire α = 0.91; arousal α = 0.92; lubrication α = 0.92; orgasm α = 0.91; satisfaction α = 0.90; pain α = 0.91). Therefore, the reliability of the FSFI is established.

### 3.3 Construct validity

As described in Section 2.3, the assumptions of confirmatory factor analyses were met, and we proceeded with the analyses. The CFA results are presented in Table 4.

**TABLE 4:** Results of the confirmatory factor analyses

	χ^2^	χ^2^/df	TLI	CFI	RMSEA	AIC	SRMR	Factor loading range	Item communalities
Model 1: Second‐order six‐factor	347.06 df = 146 *p* < 0.001	2.38	0.90	0.91	0.10	473.06	0.08	First‐order: 0.80–0.94 Second‐order: 0.50–0.96	First‐order: 0.64–0.88 Second‐order: 0.25–0.92
Model 2: Six‐factor	296.29 df = 137 *p* < 0.001	2.16	0.91	0.93	0.09	440.29	0.05	0.80–0.95	0.64–0.90
Model 3: Five‐factor	375.01 df = 142 *p* < 0.001	2.64	0.88	0.90	0.11	509.01	0.06	0.76–0.94	0.63–0.87

Abbreviations: AIC, Akaike information criterion; CFI, comparative fit index; RMSEA, root mean square error of approximation; SRMR, standardized root mean square residual; TLI, Tucker–Lewis index.

**FIGURE 1:**
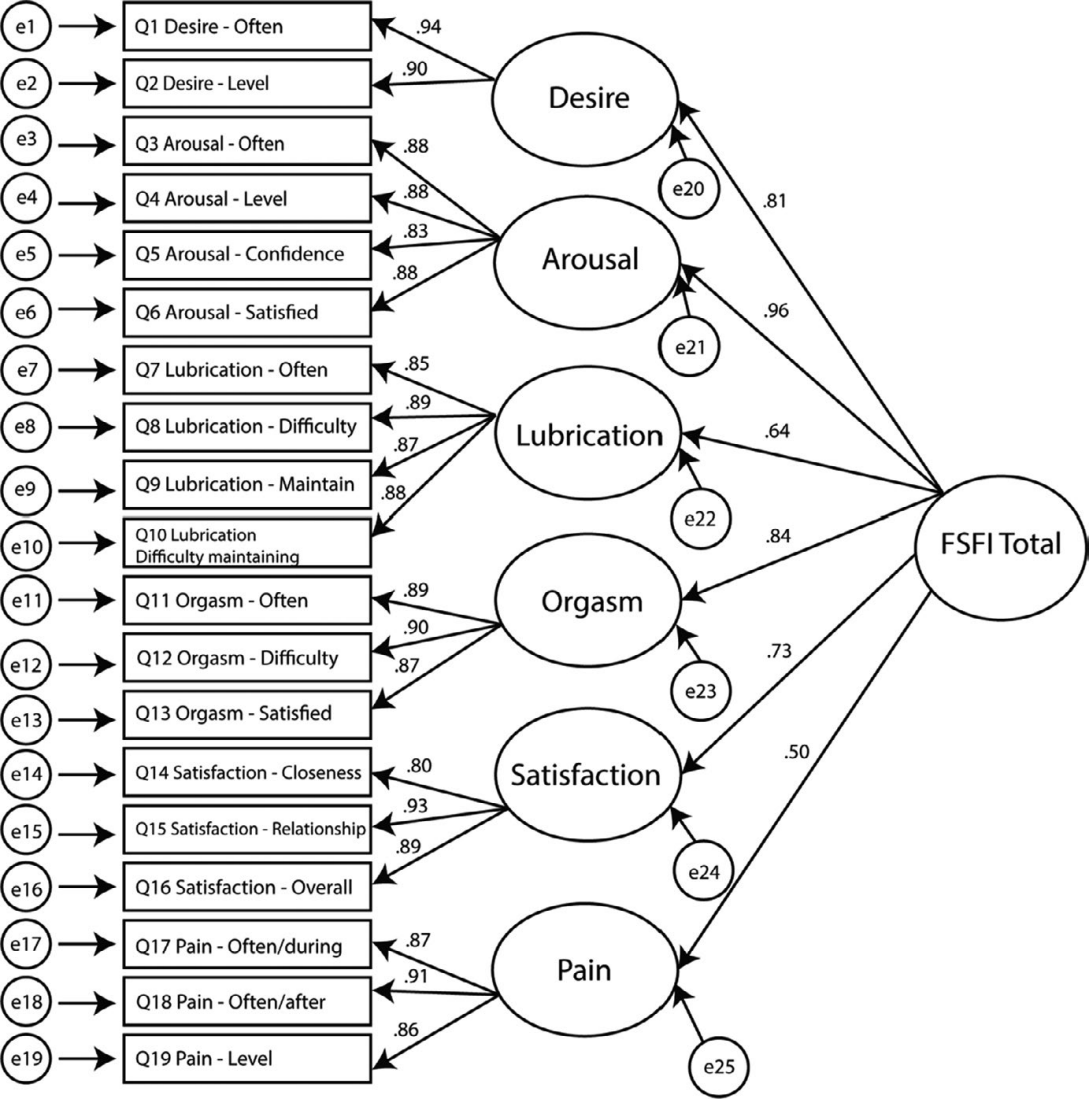
Second‐order six‐factor FSFI model with item factor loadings and subscale correlations. FSFI, Female Sexual Function Index

Model 1 (Figure 1) demonstrated marginal fit on most indices, but a poor fit on the RMSEA. All items had high factor loadings onto their relevant first‐order constructs, but some second‐order constructs did not load as strongly onto the composite total score (e.g., lubrication and pain subscales, see Figure 1).

**FIGURE 2:**
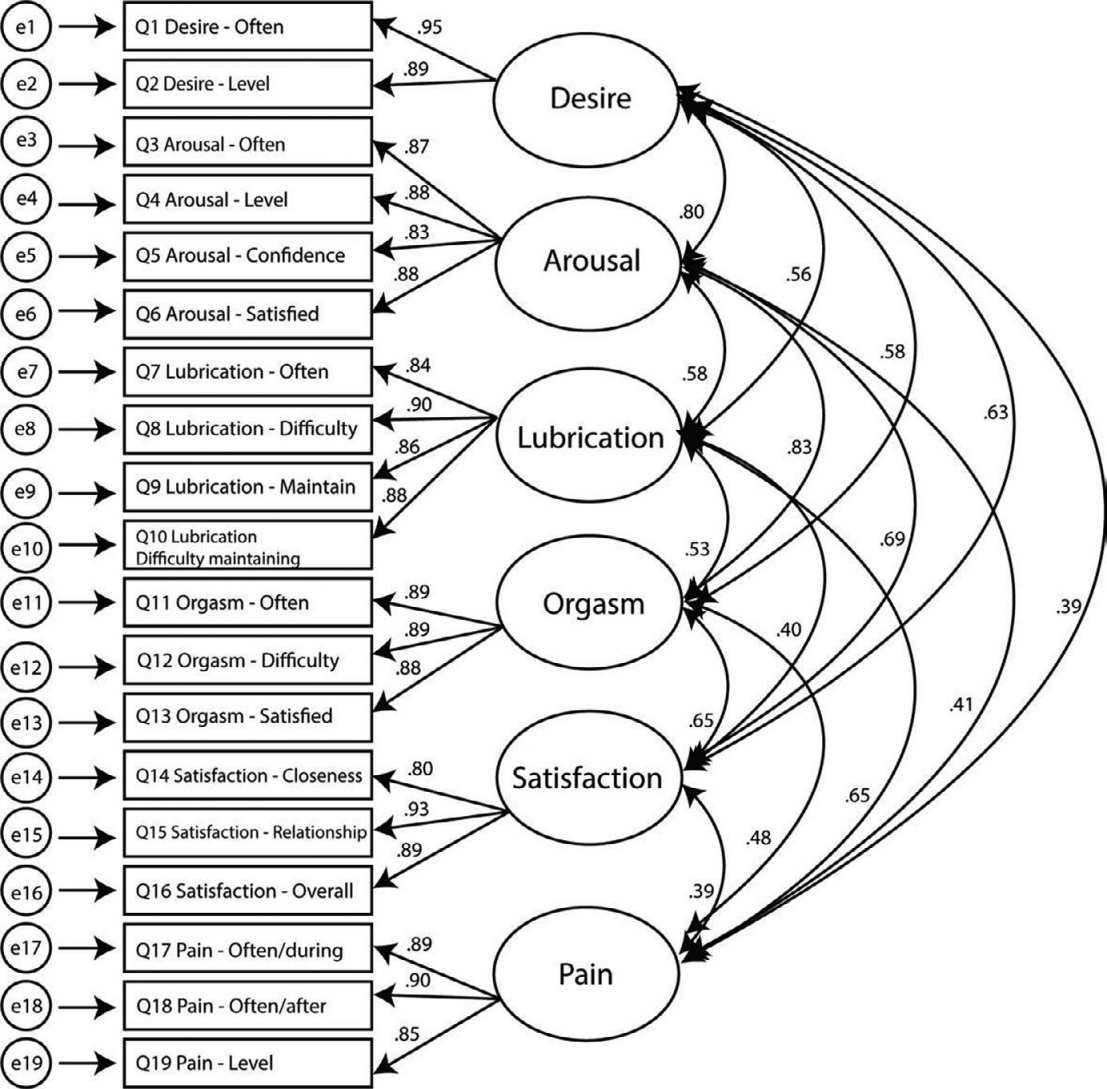
Six‐factor FSFI model with item factor loadings and subscale correlations. FSFI, Female Sexual Function Index

Model 2 (Figure 2) demonstrated superior fit compared to all other models with the smallest AIC. However, CFA results reveal a marginal fit for this model: while the χ^2^/df criteria and SRMR indicate a good fit, the TLI and CFI support a marginal fit, and the RMSEA indicates a poor fit. All items had high factor loadings (Figure 2), and correlations among all subscales were all significant (Figure 2, *p*s < 0.001). The average item communalities (Table 5) indicate that the six‐factor model explained 77% of the item variance.

**TABLE 5:** Squared multiple correlations (communalities), composite reliability (CR) values, and average variance explained (AVE) values for Model 2: Six‐factor model of the FSFI

Construct	Item	Squared multiple correlations (communalities)	Composite reliability (CR)	Average variance explained (AVE)
Desire	1	0.90	0.91	0.84
	2	0.79		
Arousal	3	0.76	0.92	0.75
	4	0.77		
	5	0.69		
	6	0.78		
Lubrication	7	0.71	0.93	0.76
	8	0.81		
	9	0.74		
	10	0.77		
Orgasm	11	0.79	0.92	0.79
	12	0.79		
	13	0.77		
Satisfaction	14	0.64	0.91	0.77
	15	0.87		
	16	0.80		
Pain	17	0.80	0.91	0.77
	18	0.80		
	19	0.72		

Abbreviation: FSFI, Female Sexual Function Index.

**FIGURE 3:**
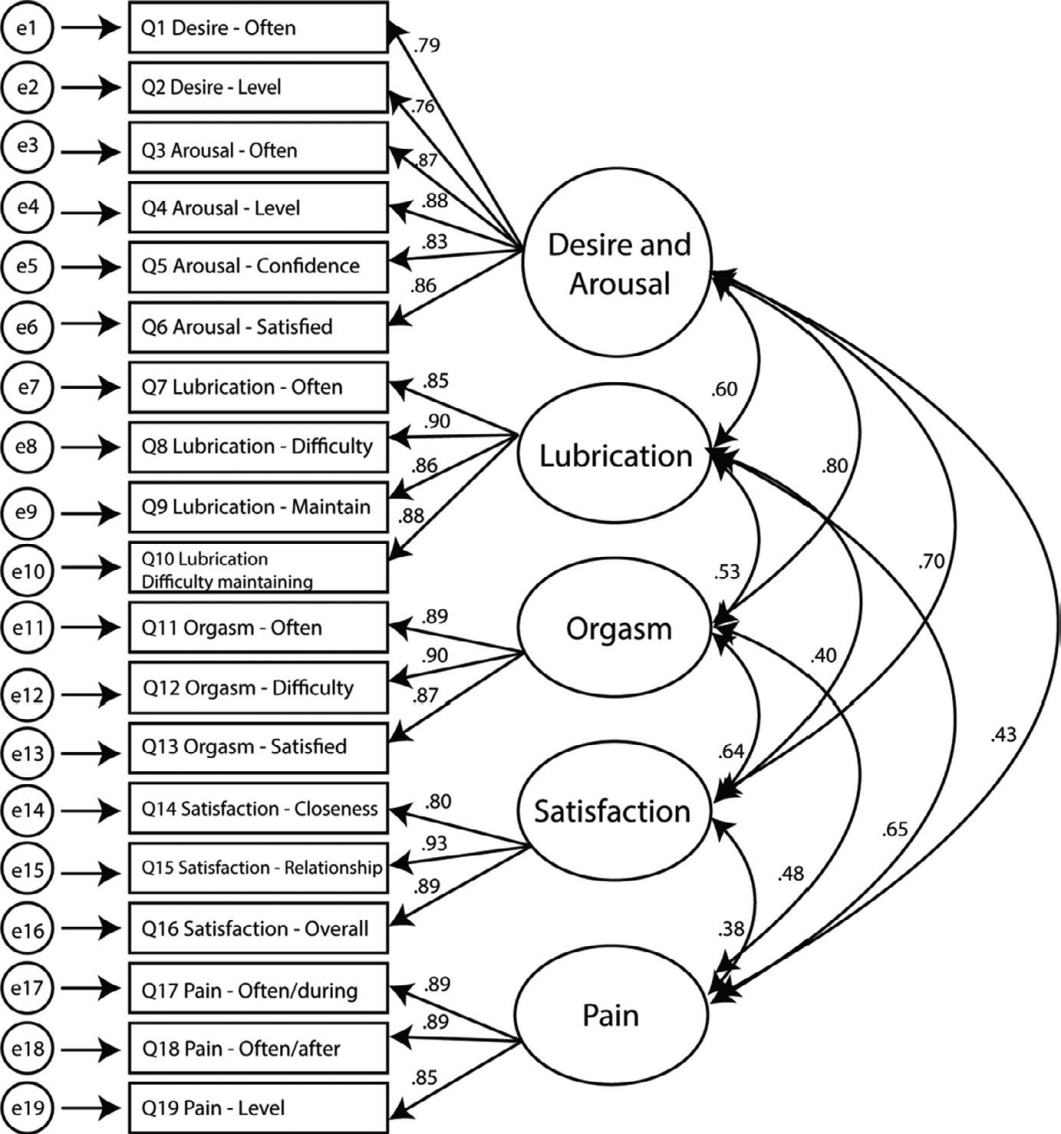
Five‐factor FSFI model with item factor loadings and subscale correlations. FSFI, Female Sexual Function Index

Model 3 (Figure 3) demonstrated marginal fit on most indices, but a poor fit on the TLI and RMSEA. All items had high factor loadings (Figure 3), and correlations among all subscales were all significant (Figure 3, *p*s < 0.001). The average item communalities indicate that the five‐factor model explained 75% of the item variance.

### 3.4 Convergent validity

Convergent validity is the extent to which: (a) a latent variable represents a construct,^28^ and (b) a measure loads onto the hypothesized construct.^29^ Here, it is the verification of six distinct constructs that represent the six domains of sexual functioning. As such, there are two criteria for convergent validity. First, the average variance extracted (AVE), as seen in Equation (1), must be greater than 0.50. The AVE is the amount of common variance among observed variables within a construct, and values >0.50 indicate that >50% of the variance in a measure is due to the hypothesized construct,^30^

(1) $$\frac{{\left(\sum \text{Factor loading value}\right)}^{2}}{{\left(\sum \text{Factor loading value}\right)}^{2}+\left(\sum \text{Measurement error}\right)}.$$

Second, the composite reliability (CR) score, as seen in Equation (2), must be >0.70.^31^ CR is the conventional notion of reliability, and it measures the total amount of true variance in relation to the total variance for each construct,

(2) $$\frac{\sum {\text{Factor loading value}}^{2}}{\sum {\text{Factor loading value}}^{2}+\sum \text{Measurement error}}.$$

From Table 5, all AVE values are >0.50, and all CR values are >0.70. Therefore, the convergent validity of the FSFI is established.

### 3.5 Discriminant validity

Discriminant validity indicates whether latent variables can be distinguished from each other.^28^ Here, the six constructs of sexual function must be distinct variables. As such, there are two criteria for discriminant validity. First, correlations between constructs should be <1.00.^29^ Second, the AVE values for each construct should be greater than its shared variance with any other construct (*r* ^2^).^30^

All construct correlations are <1.00 (Figure 2), and the AVE values for all constructs are greater than the shared variance (*r* ^2^) between all constructs (Table 6). Therefore, the discriminant validity of the FSFI is established.

**TABLE 6:** Shared common variance (*r* ^2^)

Construct	Desire	Arousal	Lubrication	Orgasm	Satisfaction	Pain
Desire	‐					
Arousal	0.64	‐				
Lubrication	0.31	0.34	‐			
Orgasm	0.33	0.69	0.28	‐		
Satisfaction	0.39	0.48	0.16	0.42	‐	
Pain	0.15	0.17	0.42	0.23	0.15	‐

Note

All values significant to *p* < 0.001.

### 3.6 Exploratory factor analyses

Given the imperfect fit of the three models evaluated in the CFA, we performed an exploratory factor analysis (EFA) to further investigate the underlying factor structure. Principle Axis Factoring was used as the extraction method with an oblique (direct oblimin) rotation method, as these methods allow for correlated factors. Kaiser's criterion and a scree plot were used as data‐driven stopping rules. The item communalities (Table 7) demonstrated high item variance accounted for by the model. Following extraction, four factors were retained. All four factors explained 73% of the variance in the data, with individual factor contributions displayed in Table 7. Additionally, Table 7 also demonstrates the extracted factor loadings, with loadings <0.40 suppressed in this model. Loadings >0.40 were weak but acceptable, with loadings >0.70 considered strong.

**TABLE 7:** Rotated factor loadings and extracted communalities from all items

Construct	Item	Factor 1	Factor 2	Factor 3	Factor 4	Communalities
Desire	1			−0.41		0.64
	2					0.56
Arousal	3	0.63				0.72
	4	0.62				0.73
	5	0.58				0.65
	6	0.74				0.79
Lubrication	7			−0.81		0.74
	8			−0.80		0.80
	9			−0.76		0.71
	10			−0.82		0.73
Orgasm	11	0.95				0.76
	12	0.80				0.75
	13	0.79				0.70
Satisfaction	14				0.76	0.59
	15				1.03	0.95
	16				0.70	0.77
Pain	17		0.72			0.80
	18		0.79			0.77
	19		0.67			0.66
Eigenvalues		9.89	2.47	1.34	1.13	
Variance explained (%)		50.59	11.68	5.79	4.59	

Thus, four distinct factors underlie the FSFI in this sample. The factor correlations were moderate to high (*r* = 0.15–0.63), consistent with the CFA results indicating correlated factors. Factor 1 contained all arousal and orgasm items, suggesting a “sexual response” latent variable. Factor 2 contained all items consistent with the pain subscale. Factor 3 contained all lubrication items and desire item 1 (weak loading), largely consistent with the lubrication subscale. Finally, Factor 4 contained all items consistent with the satisfaction subscale.

## 4 DISCUSSION

This analysis aimed to determine the reliability and validity of the FSFI in our sample of 132 sexually active women previously treated for breast cancer (noting that 125 women in the sample indicated they were not sexually active). Additionally, a CFA examined three competing models: (a) second‐order six‐factor, (b) six‐factor, and (c) five‐factor (merged arousal and desire) models. Our results demonstrate that the FSFI total and domain scores had excellent reliability, with convergent and discriminant validity also established in this population. CFA results revealed that although the six‐factor model provided the best fit of the three models evaluated, none of the tested models demonstrated a good fit across all fit indices. This result is inconsistent with previous CFA investigations of the FSFI, where evidence typically supports a good fit for a six‐factor model,^6^, ^11^, ^32^, ^33^, ^34^ or a five‐factor model with merged desire and arousal domains.^5^, ^31^, ^35^ However, in line with our findings, several of these previous CFA investigations have reported poor model fit for a six‐factor model prior to conducting exploratory modifications to improve model fit, such as removing Item 14 (satisfaction with emotional closeness),^6^ adding latent variables to describe item valence,^36^ and allowing several error terms to covariate.^37^, ^38^ Given that these model modifications do not necessarily have a priori theoretical underpinnings, we proceeded to further explore our data through an EFA to extract the underlying factor structure for comparison with known models.

Our EFA supported a four‐factor model, with an arousal/orgasm domain, and the original pain, lubrication, and satisfaction domains. Desire Item 1 loaded weakly onto the lubrication factor, and desire Item 2 did not load onto any factor. The overlap between the lubrication factor and desire Item 1 could be due to the high intercorrelations between these domains as seen in the CFA (*r* = 0.56; 31% shared common variance). This is consistent with evidence indicating that the strongest predictor of lubrication difficulties was self‐reported deficits in sexual desire.^39^ However, as the desire items did not load strongly onto any factor, we did not observe a desire factor in our sample.

Support for FSFI models with less than five factors is not uncommon. Neijenhuijs et al. reported that several investigations (mostly principal component analyses [PCA]) demonstrated support for a less‐than‐five‐factor model.^7^ However, none of these investigations report a factor structure that aligned with our results. Our lack of a distinct desire factor is also inconsistent with previous studies’ reporting of desire either as a distinct factor,^40^, ^41^, ^42^ or merged with arousal.^5^, ^8^, ^16^, ^35^ While early FSFI validation studies found greater statistical support for merged desire/arousal domains,^5^, ^16^ they were separated based on clinical considerations to allow greater treatment specificity. Some authors further argue for the desire factor to be clearly distinguished as a separate domain as it provides valuable insight into concerns regarding female sexual functioning, proposing that the desire items could be rephrased to better reflect the complexity of how desire manifests in women.^16^, ^37^

A possible explanation for the lack of a distinct desire factor in our sample could be that the desire latent factor was too weak to exert influence on the set of items entered into the EFA, given the small factor loadings of the two desire items.^43^ This can arise due to measurement error, typically as a consequence of low reliability or inaccurate item wording, resulting in a smaller‐than‐expected amount of common variance between the desire items.^44^ While our desire subscale had excellent reliability (Cronbach's α = 0.91), with normally distributed scores that were not significantly skewed or kurtosed, it is recognized in the broader literature that using two items to measure an underlying construct is problematic.^45^ Scale creators are typically advised to include at least three items per measure of a construct to reduce uncertainty and measurement error.^45^ Although this result is inconsistent with previous research in similar populations,^6^, ^8^ it is possible that our sample contained high levels of measurement error on the desire items, resulting in a desire factor that was too weak to emerge.

There are two possible reasons for the presence of measurement error. First, our participants completed the FSFI at the very end of a large battery of surveys. As such, FSFI scores could have been affected by fatigue or survey burden. Second, the experience of sexual desire in our sample could differ from other similar samples, resulting in the absence of an underlying desire factor stemming from measurement error as described above. While desire has emerged as a factor in previous investigations of female sexual functioning in cancer populations, such as Bartula and Sherman's breast cancer sample,^6^ and Baser et al.’s oncological sample,^8^ one key difference is that our sample was recruited to participate in an intervention designed to address quality‐of‐life health needs after cancer treatment, in which sexual function concerns were addressed. Therefore, the possibility of selection bias cannot be excluded from our sample. Specifically, women who self‐identify issues with their sexual functioning might have been more inclined to participate in our study. Together, these two reasons, compounded with the issue that the desire subscale only contains two items while all other subscales consisted of three or more items, could result in the desire subscale being more sensitive to uncertainties associated with measurement error, thus giving rise to a lack of a desire factor.

Next, our data also indicate a merged arousal/orgasm factor, suggesting the possibility of a “sexual response” construct. This result is inconsistent with previous investigations in breast cancer and other cancer populations,^6^, ^8^ in which distinct arousal and orgasm factors have emerged in these analyses. However, an early FSFI validation PCA in women without a clinical diagnosis of sexual dysfunction found that some arousal and orgasm items loaded on a single factor,^16^ consistent with the high domain intercorrelations found in the aforementioned study, and in our CFA as well (*r* = 0.83; 68% shared common variance). Additionally, an EFA of an FSFI created for breast cancer patients (i.e., FSFI‐BC) found that arousal and orgasm items loaded on a single factor for sexually active participants, but not for sexually inactive participants.^46^ Thus, it is possible that breast cancer treatment‐related changes in conceptualizations of arousal and orgasm are more likely to be picked up in data‐driven FSFI investigations (e.g., EFA) as compared to theory‐driven hypothesis testing (i.e., CFA).

To explain these treatment‐related changes in “sexual response”, the female arousal and orgasm experience can be conceptualized as a rhythmic model of orgasm, where multiple recurring positive feedback loops of sexual stimulation and sexual arousal culminate in sexual climax.^47^ This model suggests that experiences of arousal and orgasm share an underlying latent factor, i.e., stimulation. Such stimulation includes both physical stimulation and mental stimulation (e.g., fantasizing). Research in breast cancer samples indicates that both aspects are important in not only helping a woman reach orgasm but also in maintaining intimate relationships with their partners.^48^ As such, the role of stimulation in female arousal and orgasm could result in an interdependent relationship between these two constructs for women who have been treated for breast cancer, which would be consistent with the findings of other FSFI validation studies where arousal and orgasm items loaded onto a single factor.^16^, ^46^

Overall, despite evidence that the FSFI has sound reliability, convergent validity, and discriminant validity in our sample, our results suggest that the current conceptualizations of the FSFI might not accurately represent sexual functioning in women previously treated for breast cancer. Consistent with investigations in a healthy sample,^11^ we did not find strong support for the second‐order six‐factor model, indicating that totaling the six domains of the FSFI into a composite total score does not adequately represent female sexual functioning. Thus, contrary to the original FSFI scoring guidelines,^5^ we do not recommend the use of the total composite score as the only indicator of overall sexual functioning. Furthermore, our other tested models demonstrated, at best, a marginal fit. Combined with the EFA results, our findings suggest that female sexual functioning in our sample is best explained by a four‐factor model with distinct domains of sexual response (i.e., arousal and orgasm), lubrication, pain, and satisfaction. However, the clinical relevance of this four‐factor model, including the minimal clinical important difference (i.e., the smallest difference in score that patients perceive as beneficial), remains to be tested before it can be recommended for clinical or research use. Theoretically, while our findings provide parsimony in the conceptualization of the FSFI by reducing the constructs of arousal and orgasm to a single sexual response factor, further investigations are necessary to ascertain the role of desire in female sexual functioning. In particular, it would be prudent to consider rephrasing the current desire items to better represent the complexity of female sexual desire,^37^ and also to generate more desire items to reduce measurement error and better assess this construct.^45^

It is important to note that our inferences are limited as our sample was not screened for the presence of sexual activity in the preceding month. This led to reduced statistical power as a conservative approach was utilized to determine sexual activity, where participants who provided a zero response on any item were classified as sexually inactive and excluded from analyses. While this approach allowed for valid measurements of true sexually active respondents, it could have missed several participants who did not engage in traditional penetrative sexual intercourse. Additionally, almost 40% of sexually inactive women in our sample were separated, divorced, widowed, or single, as compared to less than 8% of sexually active women. This indicates that, while one does not need to have a partner to be sexually active, partner contributions (or lack thereof) can influence whether women engage in sexual activity after breast cancer treatments, and thus should also be considered in clinical and research contexts.

The above limitation constitutes a highly debated and controversial problem with the FSFI’s scoring system, regarding its lack of sensitivity in assessing the sexual function of women who have not engaged in sexual activity in the preceding month. In clinical assessment, interpreting zero scores as the highest degree of sexual dysfunction could incorrectly gauge a woman as having a sexual function disorder, when none was present. In research, zero scores could reduce the utility and validity of the FSFI, as conceptually, not participating in sexual activity does not necessarily indicate dysfunction. Furthermore, this scale does not capture conditions in which sexual inactivity was a result of life circumstances independent of sexual dysfunction, such as partner contributions,^49^ or the woman's pre‐cancer sexual functioning.^1^

Bartula and Sherman present a solution to this issue. They adapted the FSFI to more closely represent the sexual functioning concerns reported by breast cancer patients and thereby created the FSFI‐BC.^46^ The FSFI‐BC contains seven subscales, with three subscales assessing changes after cancer, satisfaction, and distress in sexually active and inactive women, and four subscales assessing desire/arousal, lubrication, orgasm, and pain in the sexually active group, with the same subscales but assessing the reasons for sexual inactivity in the sexually inactive group. The FSFI‐BC also includes four items exploring the partner's contributions (for clinical use only), and these items do not contribute to the total FSFI‐BC score. The FSFI‐BC has sound psychometric properties and a high degree of acceptability to participants. This adapted scale demonstrates excellent progress in the field by measuring partner contributions, pre‐cancer functioning, and sexual functioning‐specific distress across sexually active and inactive women. However, some of the new subscales included do not load onto a single factor, and the arousal‐orgasm items are merged into one factor for sexually active women, but remain distinct in sexually inactive women, further complicating the structural validity of the FSFI‐BC. Additionally, while the partner items provide useful qualitative data for clinical use, more work is needed to incorporate these items into the scale itself to quantify the influence of partner contributions on female sexual functioning. The theoretical underpinnings of the FSFI‐BC remain to be tested as well, such as the implications of merging the desire and arousal domains, and the merged arousal‐orgasm factor in sexually active women.

In conclusion, this study presents a novel four‐factor model of the FSFI, providing insights into the perspectives of sexually active women after breast cancer treatment. These insights include the importance of physical and mental stimulation underlying sexual response (i.e., arousal and orgasm). However, the FSFI does not reliably capture changes if the respondent has not engaged in sexual activity in the preceding month, or if the desire subscale is subject to measurement error. This could limit its ability to capture and monitor sexual function changes during cancer treatment or recovery in clinical or research populations. While the FSFI‐BC provides a good solution to this problem,^46^ it is important to elucidate the factors that influence sexual functioning, especially desire, arousal, and orgasm in sexually active women, and the reasons underlying sexual inactivity. Further investigations in cancer populations are warranted to examine the theoretical and practical underpinnings of female sexual functioning during and after cancer treatment. One suggestion is, given the inconsistencies in the results within the oncology space, that further factor analyses conducted with different samples might shed light on the FSFI performance in this population.

## CONFLICT OF INTEREST

No conflicts of interest.

## AUTHOR CONTRIBUTIONS

**Genevieve A. Kieseker:** Conceptualization, formal analysis, investigation, methodology, software, visualization, writing – original draft. **Alexandra L. McCarthy:** Conceptualization, project administration, validation, resources, supervision, writing – review and editing. **Debra J. Anderson:** Funding acquisition, writing – review and editing. **Janine Porter‐Steele:** Conceptualization, data curation, supervision, validation, writing – review and editing.

## Data Availability

The data that support the findings of this study are available on request from the corresponding author. The data are not publicly available due to privacy or ethical restrictions.
